# Sweet syndrome following Moderna COVID-19 vaccine: A case report

**DOI:** 10.1177/2050313X221117884

**Published:** 2022-08-11

**Authors:** Frederic Pelchat, Cynthia Fournier, Emilie Perron, Martin Gilbert, Bernard Delisle

**Affiliations:** 1 Department of Dermatology, CHU de Québec, Laval University, Québec, QC, Canada; 2 Department of Pathology, CHU de Québec, Laval University, Québec, QC, Canada

**Keywords:** Sweet syndrome, COVID-19 vaccine, Moderna vaccine, COVID vaccine

## Abstract

With the COVID-19 pandemic, vaccines have been administered all around the world probably more than ever. Even though they are considered safe, with such a huge quantity of doses used, many adverse effects including cutaneous ones were reported. We report here the case of a male adult with a history of monoclonal gammopathy of undetermined significance who presented with an extensive cutaneous eruption of indurated erythematous papules and plaques 2 days after receiving his first dose of Moderna COVID-19 vaccine (mRNA-1273 SARS-CoV-2 vaccine). Histopathology was compatible with a histiocytoid Sweet syndrome and history suggested that the vaccine contributed to the eruption.

## Introduction

Sweet syndrome, also called acute febrile neutrophilic dermatosis, is an uncommon inflammatory disease.^1^ Characteristic cutaneous lesions consist of edematous, erythematous, and tender papules and plaques that can occur on any part of the body but favor the head, neck, and upper extremities.^1^ Skin manifestations are often accompanied by systemic symptoms like fever, malaise, myalgias, and arthralgias.^1^ Leukocytosis with neutrophilia and elevated inflammatory markers are frequent laboratory findings.^1^ Usually, a Sweet syndrome diagnostic imposes a medical workup, and in at least half of the patients, a trigger or an associated disorder can be found.^1^ Malignancies, infections, drugs, autoimmune, and gastrointestinal disorders are the most common associations.^1^ Cases of Sweet syndrome have been reported following some vaccinations such as influenza and pneumococcal.^2–9^

COVID-19 pandemic made it necessary and urgent for pharmaceutical companies to develop and make accessible vaccines to help control the disease spreading on the planet. Vaccines have been and will continue to be used extensively. Thereby, adverse reactions have been reported including a variety of cutaneous ones. According to current literature, a few cases of Sweet syndrome following a COVID-19 vaccine have been described and we report one of the first histiocytoid variants of Sweet syndrome triggered by this vaccine.

## Case report

**Figure 1.**
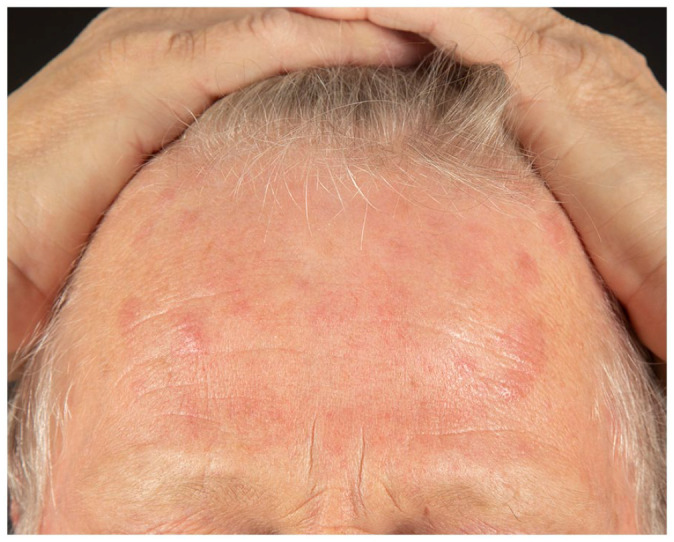
Edematous and erythematous papules and plaques on the forehead.

**Figure 2.**
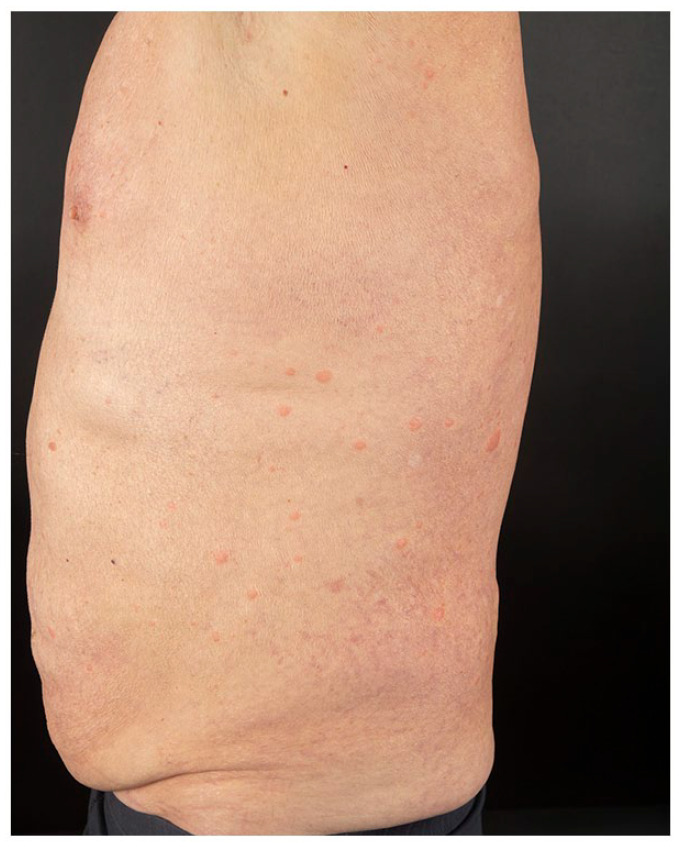
Multiple edematous, erythematous, and well-demarcated papules and plaques on the trunk.

**Figure 3.**
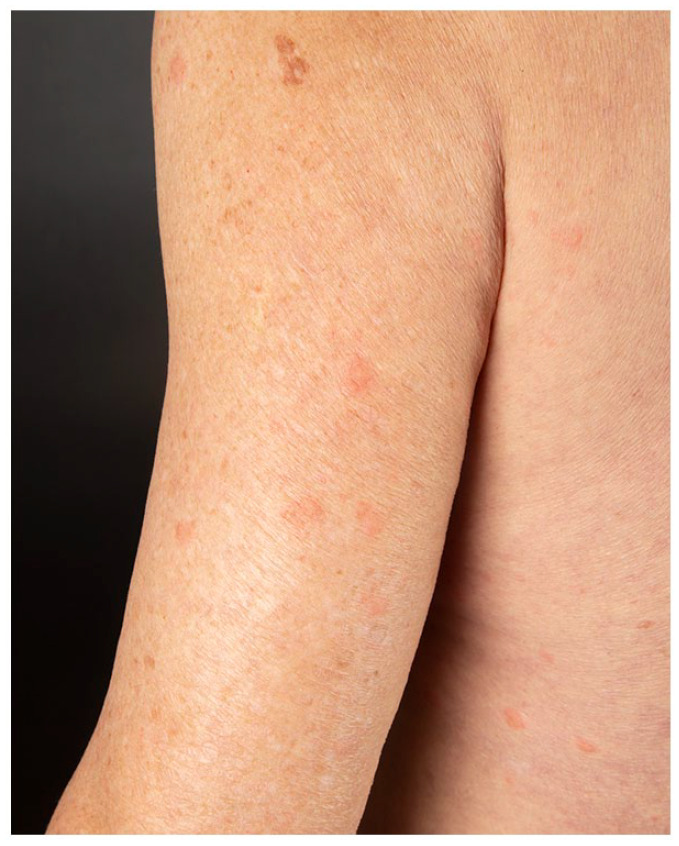
Edematous and erythematous papules of an arm.

**Figure 4.**
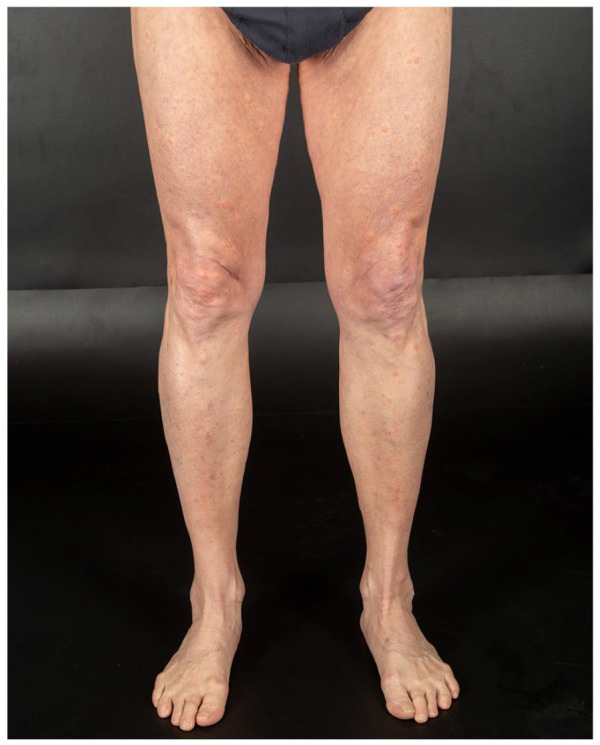
Numerous edematous, erythematous, and well-demarcated papules and plaques on the legs.

A 60-year-old male with a history of a traumatic skin burn on his back, a monoclonal gammopathy of undetermined significance and one episode of Sweet syndrome following upper respiratory tract infection presented to the emergency with a skin eruption with abrupt onset. He presented multiple, tender, indurated erythematous papules and plaques over his face, trunk, upper, and lower limbs (Figures 1–4). The eruption started 2 days after receiving his first dose of Moderna COVID-19 vaccine (mRNA-1273 SARS-CoV-2 vaccine) by intramuscular injection. Interestingly, a year before, he had the same eruption following an upper respiratory tract infection. He was seen by a dermatologist who performed a skin biopsy and histopathology was consistent with a diagnosis of classic Sweet syndrome. At this moment, he did not have a COVID-19 screening test. A large workup was made at the time of this first episode of Sweet syndrome and permitted to identify a monoclonal gammopathy of undetermined significance.

**Figure 5.**
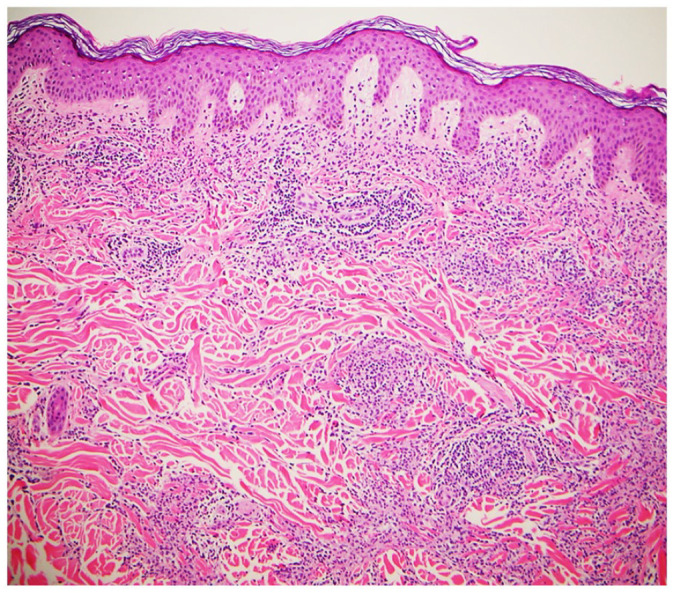
Skin biopsy show superficial and mid dermal histiocytoid cells infiltrate with a few accompanying T lymphocytes (low power view, hematoxylin and eosin stain).

**Figure 6.**
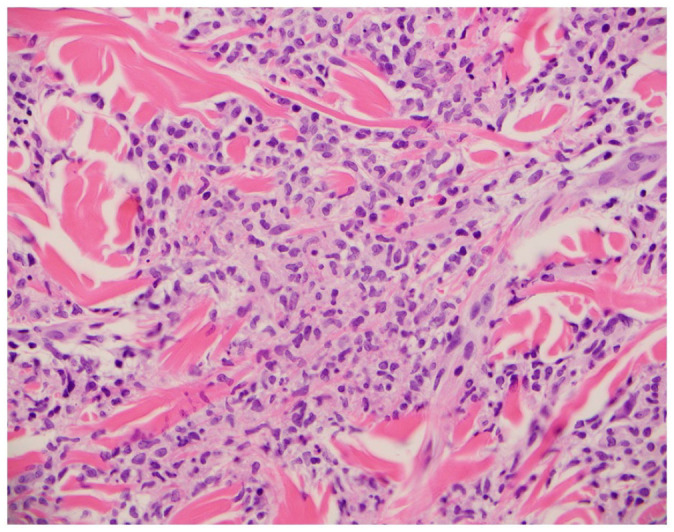
High power view of histiocytoid cells infiltrate in the dermis on hematoxylin and eosin stain.

**Figure 7.**
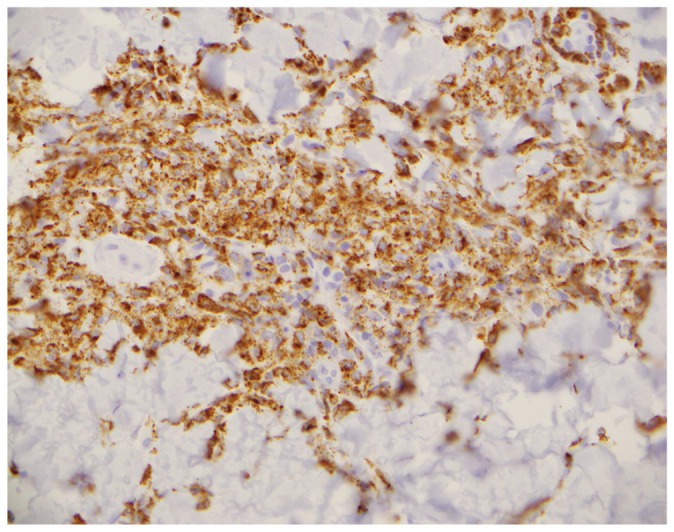
CD68 antibody staining of histiocytoid cells.

**Figure 8.**
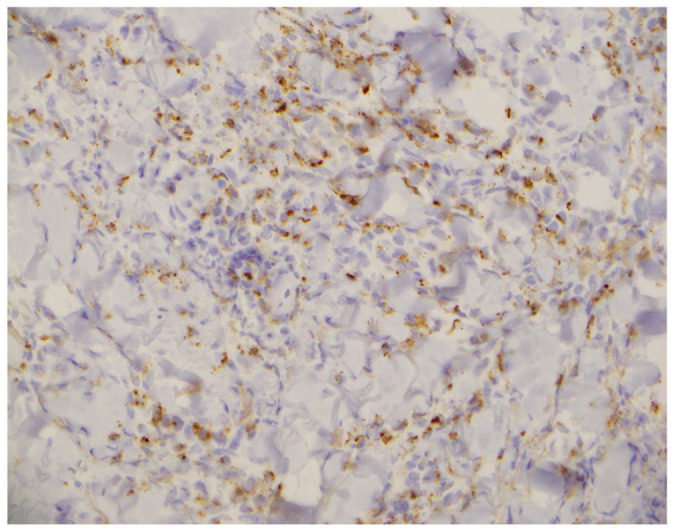
Myeloperoxidase (MPO) antibody staining of histiocytoid cells.

There were no other systemic signs or symptoms including fever, chills, arthralgias, or myalgias. He only complained of mild malaise and fatigue. Laboratory tests revealed a mild neutrophilia at 8310 × 10^9^/L (normal range 1900–7000 × 10^9^/L) and an elevated sedimentation rate at 15 mm/h (normal range 0–10). His SARS-CoV-2 blood serology was negative. Histopathology revealed superficial and mid-dermal histiocytoid cells infiltrate with a few accompanying T lymphocytes (Figures 5 and 6). Histiocytoid cells were CD68+, CD163+, and myeloperoxidase+ (Figures 7 and 8). Skin direct immunofluorescence studies did not reveal any immunoglobulin deposit. Histopathology was compatible with histiocytoid Sweet syndrome variant.

We treated the patient with systemic corticosteroid and he had an excellent response. We continued prednisone for a few months in tapering doses since the eruption tended to recur at many attempts of stopping prednisone. The eruption gradually subsided after. Based on the patient’s history, his COVID-19 vaccine trigger, physical examination, histopathology, laboratory findings, and excellent response to systemic corticosteroid, the patient met the diagnosis criteria of Sweet syndrome following COVID-19 Moderna vaccination.

Twelve weeks after his first dose of COVID-19 vaccine, he received his second dose of the same Moderna COVID-19 vaccine. Less than 24 h later, he developed the same acute eruption of tender erythematous papules and plaques that rapidly subsided 48 h after. The patient was still on a low dose of corticosteroid. A few weeks later, he received his third dose of the same Moderna COVID-19 vaccine. He was still on a low dose of corticosteroid. He did not experience any cutaneous eruption at this time, not even when the corticosteroid was fully weaned off.

## Discussion

According to the manufacturer, Moderna COVID-19 Vaccine contains a lipid nanoparticle comprised of a messenger ribonucleic acid (mRNA) encoding the pre-fusion stabilized Spike glycoprotein of SARS-CoV-2 virus.^10^ It is indicated for active immunization against coronavirus disease 2019.

Extensive data about the COVID-19 different types of vaccines and their side effects are still to be made. Many cases of cutaneous adverse effects following vaccination have been described. They include local cutaneous reactions, urticaria, morbilliform eruptions, immune dermatoses, and other rare reactions.^11^

We presented the case of a Sweet syndrome following COVID-19 vaccination. Our patient therefore met the criteria for the diagnosis of Sweet syndrome. The short time interval between the administration of the vaccine and the cutaneous eruption allows us to identify the vaccine as the principal trigger. Sweet syndrome has been described following pneumococcal, influenza, and BCG (Bacille Calmette–Guérin) vaccination.^2–9^ To our knowledge, only a few cases of Sweet syndrome following COVID-19 vaccination have been reported to date. Four of them were associated with the Oxford-AstraZeneca vaccine.^12–15^ Two others with the mRNA Pfizer-BioNTech vaccine^16,17^ and only one^18^ with the same vaccine as our case, the Moderna mRNA01273 vaccine. The patient in this last case had a neutrophilic dermatosis with histiocytoid cells on histopathology and he also presented extra-cutaneous adverse effects attributed to the vaccine such as myoclonus and encephalitis. Vaccination seems to trigger an immune response and activate or reactivate this syndrome. Our case is one of the first reported histiocytoid variant of Sweet syndrome following a COVID-19 vaccine. Histiocytoid variant is an uncommon variant of Sweet syndrome. It is more often associated to malignancies than other variants of the disease.^19^ Our patient was known for a gammopathy of unknown significance and will be closely monitored to make sure it does not transform into a malignancy in the future.

We described the case of an uncommon dermatologic disease following COVID-19 vaccination. With the actual important use of these vaccines, it seems important to report unusual cases like this one so that data about their safety can be made.
